# Increased white blood cell count is associated with an increased demand for unfractionated heparin during veno-arterial extracorporeal oxygenation in lung transplantation

**DOI:** 10.1051/ject/2024022

**Published:** 2024-09-20

**Authors:** Koichi Kashiwa, Hideo Kurosawa, Kazuki Fujishiro, Hitoshi Kubo, Ryota Inokuchi, Masahiko Bougaki, Gaku Kawamura, Masaaki Sato, Chihiro Konoeda, Jun Nakajima, Kent Doi

**Affiliations:** 1 Department of Clinical Engineering, The University of Tokyo Hospital 7-3-1, Hongo Bunkyo-city, Tokyo 113-8655 Japan; 2 Department of Anesthesiology, The University of Tokyo Hospital 7-3-1, Hongo Bunkyo-city, Tokyo 113-8655 Japan; 3 Department of Thoracic Surgery, The University of Tokyo Hospital 7-3-1, Hongo Bunkyo-city, Tokyo 113-8655 Japan

**Keywords:** Veno-arterial extracorporeal membrane oxygenation, Lung transplantation, Unfractionated heparin, White blood cell

## Abstract

*Background*: This retrospective observational study aimed to examine whether clinical inflammatory parameters were associated with the requirement dosage of unfractionated heparin (UFH) to maintain the range of ACT in veno-arterial extracorporeal membrane oxygenation (V-A ECMO) during lung transplantation surgery. *Methods*: Among all patients who underwent lung transplantation using V-A ECMO from January 2021 to May 2022, 27 patients were included. These patients were divided into two groups based on whether the infusion rate of UFH was increased from the initial infusion rate (7–8 units/kg/h) (increased group, *n* = 10) or the infusion rate was maintained or decreased (non-increased group, *n* = 17). The infusion rate was adjusted with an activated clotting time (ACT) target of 160–200 s. *Results*: At 1–2 h after starting ECMO, ACT was significantly lower (179.0 (166.5–188.5) versus 224.0 (193.0–242.0) sec, *p* = 0.006) and white blood cell (WBC) counts were higher in the increased group (12.6 ± 3.3 versus 9.5 ± 4.0 × 10^3^/μL, *p* = 0.046). The UFH infusion rates were higher in the increased group during the surgery. The cutoff value of WBC count at 1–2 h after starting ECMO for discriminating the need for increasing the UFH dosage was determined as 10.2 × 10^3^/μL (sensitivity 90.0%, specificity 58.8%, area under the curve 0.712) and discrimination of this cut-off value was confirmed as statistically significant (*p* = 0.018). *Conclusion*: These data suggested that WBC count was associated with the requirement of an increase in the UFH infusion rate of V-A ECMO during lung transplantation surgery. Further evaluation is necessary to clarify the role of WBC count in determining the optimal UFH dosage.

## Introduction

Veno-arterial extracorporeal membrane oxygenation (V-A ECMO) is mainly used during lung transplantation when both circulatory and respiratory supports are required because of pulmonary hypertension or difficulty in maintaining oxygenation, ventilation, and circulation [1, 2]. Appropriate anticoagulant therapy is required during V-A ECMO, and unfractionated heparin (UFH) is most used as an anticoagulant in ECMO [3, 4]. The dosage of anticoagulants for V-A ECMO performed in the ICU is determined based on parameters such as activated partial thromboplastin time (APTT), activated clotting time (ACT), and anti-Xa activity [5–7].

It is widely recognized that inflammation has a significant effect on the blood coagulation system [8–10]. Therefore, it is necessary to pay close attention to the changes in the data associated with inflammatory reactions during ECMO. Indeed, in the ICU settings, many reports indicate issues that should be noted in the management of ECMO such as inflammatory status [11, 12]. However, few reports suggested specific anticoagulant strategies for ECMO during lung transplantation. In this retrospective study, we hypothesized that UFH dosage would increase with the increase of white blood cell (WBC) count because of the interaction between inflammation and coagulation disorder. We clinically adjusted the UFH infusion rate in V-A ECMO during lung transplantation based on the results of ACT with the target value of 160–200 s. The purpose of this study was to examine whether clinical inflammatory parameters were associated with the required dosage of UFH to maintain this range of ACT in V-A ECMO during lung transplantation.

## Materials and methods

The present retrospective study was conducted with the approval of the Ethics Committee of the University of Tokyo Graduate School of Medicine/School of Medicine (Approved No.11535).

### Cases

Among all patients who underwent lung transplantation using V-A ECMO from January 2021 to May 2022, 27 patients who were weaned from V-A ECMO during surgery were included in this study. Of the 27 patients, 7 received single-lung transplantation and 20 received bilateral lung transplantation. In two cases of bilateral lung transplantation, V-A ECMO was initiated after perfusion of the first lung. In the other cases, V-A ECMO was started before implantation of the first lung.

### ECMO management

A MERA centrifugal pump (Senko Medical Industry Co., Ltd.) and HPO-23WH-C oxygenator (Senko Medical Industry Co., Ltd.) were incorporated into the ECMO circuit. The ECMO circuit was primed with approximately 500 ml of lactated Ringer’s solution containing 1 unit/ml of unfractionated heparin (UFH). 2000 units of UFH were administered at the time of cannulation for the initial dose, and the UFH infusion was started at the rate of 7–8 units/kg/h after initiating ECMO. An ACT was measured once every 30–60 min, and the infusion rate was adjusted with a target of 160–200 s. If ACT was below 150 or 160 s, the UFH infusion rate was increased by 1 or 2 unit/kg/hr, respectively. Otherwise, the UFH infusion rate was not changed. Other parameters were not taken into consideration to adjust the infusion rate of UFH. A Hemocron Jr. Signature + , ACT-LR (ITC) was used for ACT measurement. The flow rate of V-A ECMO was based on 2.4 L/min/m^2^ and maintained parameters such as arterial pressure, pulmonary artery pressure, and blood gas data within normal limits. Blood gases were measured once every 30–60 min, and white blood cell (WBC) and platelet counts were measured via Celltac (Nihon Kohden Kogyo Co., Ltd.) at the same time points. Body temperature was maintained within normal range in all the cases.

### Examination methods

The enrolled patients were divided into two groups based on whether the infusion rate of UFH was increased from the initial infusion rate (increased group) or the infusion rate was maintained or decreased (non-increased group). Patients’ data were evaluated at the following time points: before starting ECMO, immediately after starting ECMO, 1–2 h after starting ECMO, after reperfusion of one lung, and after reperfusion of the second lung in the case of bilateral lung transplantation. These data were retrospectively collected from their medical records.

### Statistical analysis

Values are shown as mean ± standard deviation or median (interquartile range) depending on data distribution. Student’s t-test and Wilcoxon’s rank sum test were used for comparison between the two groups, and *p* < 0.05 was considered significant. Fisher’s exact test was performed for categorical data. Receiver operating characteristic (ROC) analysis was performed to evaluate the performance of discrimination. The cutoff value was determined by the Youden index. Data were analyzed using JMP Pro17 (SAS Institute, Cary, NC, USA).

## Results

### Patients’ characteristics and V-A ECMO performance

Of the 27 patients 10 were categorized into the increased group, and 17 into the non-increased group. None of the patients in the increased group needed to have a reduction in their UFH infusion rate. Correspondingly none of the patients in the non-increased group required to increase the UFH infusion rate. Two patients in whom V-A ECMO was introduced after reperfusion of the first lung were classified into the non-increase group because the UFH infusion rate after starting ECMO was not increased. As shown in Table 1, no statistically significant difference was observed between the two groups in height, weight, body surface area, preoperative hematocrit, platelet count, fibrinogen concentration, WBC count, C-reaction protein (CRP), albumin and activated partial thromboplastin time (APTT), preoperative use of immunosuppressants. Bilateral lung transplantation was performed in 90.0% of the increased group and 64.7% in the non-increased group. The duration of ECMO was significantly longer in the increased group compared with the non-increased group (314.2 ± 48.4 versus 257.9 ± 70.5 min, *p* = 0.035). The lung diseases in each group are shown in Table 2. There was no evident difference in the distribution of lung diseases between the groups.

**Table 1:** Patients’ characteristics, laboratory data, drug, intraoperative data.

	Increased group (*n* = 10)	Non-increased group (*n* = 17)	*p* value
Height (cm)	160.1 (155.3–170.6)	160.2 (155.2–165.3)	0.451
Weight (kg)	49.5 ± 15.4	48.0 ± 14.9	0.817
BSA (m^2^)	1.48 ± 0.25	1.44 ± 0.27	0.688
Preoperative Hct (%)	38.3 ± 4.9	41.2 ± 6.4	0.234
Preoperative Plt (×10^3^/μL)	319 ± 97	274 ± 125	0.338
Preoperative Fbg (mg/dL)	464.4 ± 136.9	430.3 ± 108.0	0.480
Preoperative WBC (×10^3^/μL)	9.2 (8.0–10.8)	7.9 (7.2–9.4)	0.138
Preoperative CRP (mg/dL)	1.28 (0.88–1.77)	0.49 (0.13–1.87)	0.108
Preoperative Albumin (g/dL)	3.8 (3.5–4.2)	4.2 (3.7–4.4)	0.243
Preoperative APTT (sec)	28.5 ± 5.1	27.2 ± 3.2	0.450
Preoperative use of immunosuppressants (%)	70.0	58.8	0.692
Number of double lung transplantation	9	10	0.204
ECMO duration (min)	314.2 ± 48.4	257.9 ± 70.5	0.035
Time from starting ECMO to UFH infusion rate increase (min)	161.3 ± 72.7	0	<0.001

**Table 2:** Lung diseases.

	Increased group	Non-increased group
Bronchiectasis	2 (20.0%)	2 (11.8%)
Chronic GVHD after HSCT	2 (20.0%)	2 (11.8%)
Idiopathic interstitial pneumonia	2 (20.0%)	5 (29.4%)
Collagen disease interstitial pneumonia	3 (30.0%)	1 (5.9%)
Others	1 (10.0%)	7 (41.1%)

Real numbers (%).

GVHD: graft versus host disease, HSCT: hematopoietic stem cell transplantation.

### Changes in ACT value, platelet count, WBC count, and infusion rate of UFH during V-A ECMO

Because the UFH infusion rate was adjusted based on ACT in each patient, the ACT values were lower and the infusion rates of UFH were higher in the increased group during the surgery (Table 3). UFH infusion rate was increased after 161.3 ± 72.7 min from starting ECMO in the increased group (Table 1). The timing of the UFH infusion rate increases was approximately 100 min before the end of ECMO in the non-increased group. No significant difference in platelet count was observed between the two groups at any time points (Table 4). However, at 1–2 h after starting ECMO, the WBC counts were significantly higher in the increased group compared with the non-increase group (*p* = 0.046). Although there was no statistically significant difference in the WBC count at other times, the average WBC count tended to be higher in the increased group all through the surgery (Table 4). Regarding blood product transfusion, a statistically significant difference was observed only in the amount of fresh frozen plasma (FFP) after reperfusion of one lung to the end of ECMO (Table 5). The percentage of neutrophils, eosinophils, and basophils at the WBC fraction immediately after surgery was 92.1(89.9–94.7), 0.1(0–0.1), and 0.1(0.1–0.1)%, respectively.

**Table 3:** Changes in ACT, infusion rate of UFH.

Time point	ACT (sec)			Infusion rate of UFH (units/kg/h)		
	Increased group	Non-increased group	*p* value	Increased group	Non-increased group	*p* value
Immediately after starting ECMO	211.0 (193.5–265.0)	253.5 (221.5–270.5)	0.223	6.77 ± 0.94	7.80 ± 1.65	0.082
1–2 h after starting ECMO	179.0 (166.5–188.5)	224.0 (193.0–242.0)	0.006	6.88 ± 0.89	7.46 ± 2.13	0.423
After reperfusion of the first lung	163.0 (151.5–179.5)	227.0 (164.5–249.5)	0.023	8.25 ± 1.73	7.29 ± 2.13	0.242
After reperfusion of the second lung*	150.7 ± 17.1	197.5 ± 51.8	0.041	11.72 ± 2.95	7.66 ± 2.81	0.009

*Only in bilateral lung transplantation.

**Table 4:** Changes in platelet count, WBC count.

	Platelet count (×10^3^/μL)			WBC count (×10^3^/μL)		
	Increased group	Non-increased group	*p* value	Increased group	Non-increased group	*p* value
Before starting ECMO	186 ± 53	157 ± 69	0.261	8.4 (6.2–10.7)	6.8 (5.2–8.5)	0.183
Immediately after starting ECMO	162 ± 50	140 ± 58	0.342	8.4 ± 2.9	7.0 ± 2.2	0.176
1–2 h after starting ECMO	160 ± 42	129 ± 53	0.136	12.6 ± 3.3	9.5 ± 4.0	0.046
After reperfusion of the first lung	148 ± 55	138 ± 55	0.664	10.6 (7.9–15.0)	8.4 (5.8–11.5)	0.182
After reperfusion of the second lung*	124 ± 65	84 ± 32	0.139	10.3 ± 3.9	9.2 ± 5.1	0.621
After ECMO ended	88 (82–142)	111 (74–120)	0.986	10.6 ± 4.1	8.7 ± 4.2	0.264

*Only in bilateral lung transplantation.

**Table 5:** Comparison of blood transfusion volume.

	PRBC (units)			FFP (units)			Platelets (units)		
	Increased group	Non-increased group	*p* value	Increased group	Non-increased group	*p* value	Increased group	Non-increased group	*p* value
1 h after starting ECMO	0 (0–2.0)	0 (0–2.0)	0.820	0 (0–0)	0 (0–0)	0.658	0	0	NA
From 1 h after starting ECMO to reperfusion of the first lung	2.0 (0–4.0)	2.0 (0–2.0)	0.628	0 (0–2.0)	0 (0–4.0)	0.322	0 (0–0)	0	0.192
After reperfusion of the first lung to the end of ECMO	6.0 (3.0–6.0)	4.0 (2.0–5.0)	0.274	6.0 (5.5–8.5)	2.0 (2.0–8.0)	0.042	0 (0–0)	0 (0–0)	0.626
Total	10.2 ± 4.3	8.9 ± 3.7	0.414	10.0 (8.0–12.5)	10.0 (6.0–13.0)	0.799	5.0 (0–12.5)	10.0 (0–15.0)	0.503

Volume of 1 unit is about 140, 120, and 10 ml of PRBC, FFP, and Platelets, respectively in Japan.

### Relationship between change in the UFH infusion rate and WBC count

ROC analysis determined the cutoff value of WBC count at 1–2 h after starting ECMO for discriminating the necessity of an increase in UFH infusion rate was 10.2 × 10^3^/μL (sensitivity 90.0%, specificity 58.8%) with the AUC of 0.712 (95% confidence interval 0.593–0.831). Discrimination of this cutoff value was confirmed as statistically significant by Fisher’s exact test (*p* = 0.018).

## Discussion

In the present study, we found a significant association between the patient’s ECMO WBC count and the necessity to increase UFH dosage during lung transplantation with V-A ECMO. Lower ACT was observed in the increased group from 1 to 2 h after starting ECMO, indicating that more UFH was required to maintain ACT in the target range of 160–200 s. No difference was observed in platelet count at any time points. On the other hand, WBC count was significantly higher in the increased group at 1–2 h after starting ECMO and WBC counts at other timings tended to be higher in the increased group all through the surgery (Table 4). These results suggested that the WBC count or associated inflammatory status of the patient might be involved in the changes of ACT and response to the increase of UFH in V-A ECMO during lung transplantation.

Although no data for WBC fraction was available in the present study, neutrophils might be the main portion of the WBC count increase because it is well-known that neutrophils are increased during the acute phase of inflammation [13]. On the other hand, certain portions of WBC counts might contain eosinophils and basophils, which are considered not to be dominant in the situation of acute insults such as surgery [14–17]. As shown in the results, this can be also inferred from the high percentage of a fraction of neutrophils and low percentage of a fraction of eosinophils and basophils immediately after lung transplantation. In cardiac surgery using cardiopulmonary bypass, it has been reported that neutrophil elastase concentration increases during cardiopulmonary bypass [18–20]. There is another report showing an increase in neutrophil elastase during ECMO [21]. When neutrophil elastase and other substances were increased with neutrophil activation, they promote tissue factor- and factor VII-dependent coagulation reactions and inhibit the activity of tissue factor pathway inhibitors [22]. Taken together, it is plausible that in some patients, an increase in neutrophils during lung transplantation using VA-ECMO accompanies neutrophil elastase, influencing ACT and the requirement for UFH dosage.

In the present study, there was no difference in patients’ preoperative characteristics and lung disease between the increased group and the non-increased group. However, it is presumed that inflammatory response had already been enhanced preoperatively in some patients because CRP and albumin in the increased group tended to be higher and lower than the non-increased group, respectively. Besides, because some of the patients were treated with long-term immunosuppression before lung transplantation, inflammatory reactions in such patients including WBC recruitment to the peripheral circulation may be suppressed during surgery, although there was no evident difference in preoperative immunosuppression status between groups in this study.

Currently, the infusion rate of UFH is adjusted using ACT as a main indicator. Taking the result of the present study into consideration, the WBC count, rather than the platelet count, could be an additional useful reference parameter to fine-tune the dosage of UFH during VA-ECMO in lung transplantation. However, it should be noted that several confounding factors such as intensity of surgical invasion and amount of blood transfusion might have significant impacts on the association of WBC count with UFH infusion rate. Adjusting these factors by multivariable analysis was difficult because of the small sample size of this study. However, these factors might be assumed not to influence WBC counts largely because we observed significant differences in WBC counts between the groups at the early phase of 1–2 h after starting ECMO. Further investigation is necessary to clarify the cause of the increase in WBC count and the mechanism of effect on ACT.

## Limitations

The present study has several limitations. First, this is a single-center, retrospective analysis. Multicenter prospective analysis is necessary to confirm the finding obtained in the present study. Second, all the patients were treated with the same initial infusion rate of UFH and the grouping of patients (i.e., increased and non-increased) is relative to the initial dosage. However, even if the initial infusion rate of UFH was set at a higher dose, for example, we speculate a similar relationship was observed among ACT, adjustment of dosages of UFH, and WBC count. Third, we adjusted the UFH infusion rate based only on ACT this time. It remains unclear whether similar results will be obtained when adjusting the infusion rate using other parameters for monitoring coagulation. However, other laboratory tests such as APTT or Xa activity will not be useful because rapid adjustment of anticoagulant therapy is required during lung transplantation surgery. Other blood viscoelasticity test equipment that can be used as point-of-care tests will cost much for multiple measurements during surgery. Fourth, although antithrombin (AT) was also another potential confounder, AT was not measured in all the patients in this study. Finally, the sample size in this study was small and the result is preliminary. Therefore, this study is better to be positioned as a pilot study. To confirm the results, further investigations are necessary with larger sample sizes determined by sample size calculation based on this study.

## Conclusion

This retrospective observational study demonstrated that increased WBC count during lung transplantation surgery was associated with the requirement of an increase in UFH infusion rate in V-A ECMO management. The obtained findings suggested the possible impact of inflammation on anticoagulation management in ECMO during lung transplantation surgery.

## Data Availability

Data are available from the corresponding author on reasonable request and with permission of the institutional review board of the University of Tokyo.
